# Research Progress in Gas Separation Using Hollow Fiber Membrane Contactors

**DOI:** 10.3390/membranes10120380

**Published:** 2020-11-29

**Authors:** Linlin Li, Guiyang Ma, Zhen Pan, Na Zhang, Zhien Zhang

**Affiliations:** 1 College of Petroleum Engineering, Liaoning Shihua University, Fushun 113001, China; linlin20200321@163.com (L.L.); guiyangma1@163.com (G.M.); Zhenpan_fs@126.com (Z.P.); 2 Shandong Gas Marketing Branch, Sinopec Gas Company, Jinan 250000, China; NaaZhang@hotmail.com; 3 William G. Lowrie Department of Chemical and Biomolecular Engineering, The Ohio State University, Columbus, OH 43210, USA

**Keywords:** membrane contactor, gas separation, CO_2_ capture, absorbent, membrane wetting

## Abstract

In recent years, gas–liquid membrane contactors have attracted increasing attention. A membrane contactor is a device that realizes gas–liquid or liquid–liquid mass transfer without being dispersed in another phase. The membrane gas absorption method combines the advantages of chemical absorption and membrane separation, in addition to exhibiting high selectivity, modularity, and compactness. This paper introduces the operating principle and wetting mechanism of hollow membrane contactors, shows the latest research progress of membrane contactors in gas separation, especially for the removal of carbon dioxide from gas mixtures by membrane contactors, and summarizes the main aspects of membrane materials, absorbents, and membrane contactor structures. Furthermore, recommendations are provided for the existing deficiencies or unsolved problems (such as membrane wetting), and the status and progress of membrane contactors are discussed.

## 1. Introduction

Owing to the rapid economic development since the 20th century, the climate and environment have continued to deteriorate. Natural phenomena such as the greenhouse effect and acid rain are closely related to industrial production and energy use [1,2,3,4,5]. According to data from the National Oceanic and Atmospheric Administration/Earth System Research Laboratory, global CO_2_ emissions are steadily increasing, and the CO_2_ concentration in the atmosphere has reached 411 ppm, a new record high [6]. Further, the concentrations of atmospheric and other gases have increased, resulting in the greenhouse effect and the acidification of ecosystems, which has caused significant losses to the construction industry, agriculture, and public facilities. Studies have shown that fossil fuels including coal, oil, and natural gas will still dominate the energy structure in the short term [7]. Therefore, the Organization for Economic Cooperation and Development (OECD) countries and China have formulated increasingly strict regulations and laws to limit gas emissions to ensure the sustainable consumption of fossil energy [8,9]. So, the main content of this review is focused on the progress of membrane contactors in the field of gas separation, such as CO_2_ removal from coalbed methane, flue gas, and natural gas.

**Figure 1:**
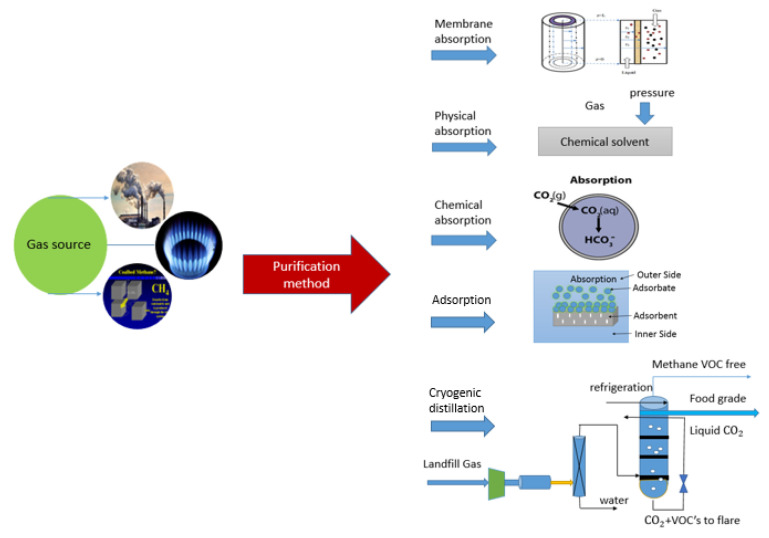
Classifications of gas treatment methods.

Advanced gas separation technology is important to satisfy the market demand for efficient and affordable sulfur emission control. Existing gas separation technologies include the physical adsorption method, low-temperature phase separation, chemical absorption, and membrane absorption methods. Traditional gas separation is primarily achieved by direct gas–liquid contact methods, such as using packed towers, spray towers, bubble towers, and other equipment, as shown in Figure 1. Membrane absorption has been developed for gas separation in recent years. A promising carbon dioxide removal process, it combines the selectivity of chemical absorption and the compactness of membrane separation. The interface area of the membrane contactor is approximately 30 times that of the conventional gas absorber, which can reduce the size of the absorber by 10 times [10]. 

Membrane materials can be primarily classified into organic polymer membranes, inorganic membranes, and organic–inorganic composite membranes. Polypropylene (PP) [11], polyvinylide–nefluoride (PVDF) [12], polytetrafluoroethylene (PTFE) [13], polysulfone (PS) [14], polyethersulfone (PES) [15], and polyimide (PI) [16] are some of the most widely used membrane materials. In recent years, ceramic membranes have become a hot research topic owing to their high temperature resistance, strong thermal and chemical stability, and low energy consumption [17], however, their cost is relatively high. In addition, researchers recently discovered that membrane modification treatment is an effective and economical method to improve membrane performance, and matrix and surface modifications are the two main methods to modify the membrane. 

The study of absorbents has always been the focus of hollow fiber membrane contactors (HFMCs) in the field of gas separation. Since the advent of membrane absorption technologies, researchers have conducted extensive studies on absorbents. Absorbents have been developed from pure water and NaOH [18] to alcohol amine [19], and gradually progressed to amino acid solutions [20], nanofluids [21], ionic fluids [22], etc. This research field is very rich, encompassing the subjects of chemistry, physics, molecular science, thermodynamics, and various other disciplines. The analysis and interpretation of some of the membrane absorption mechanisms inevitably produce numerous inconsistencies. This study points out the relevant contradictions and contrarian views. For reconciling these diverse views, more in-depth theoretical research and visual experiments aimed at understanding these mechanisms are needed.

Membrane wetting has always been a major obstacle in the development of membrane absorption methods. With an increase in the operating time, wetting of the membrane pores is often unavoidable. Even a small extent of membrane wetting has serious adverse effects on the gas separation efficiency. Researchers have attempted various methods to extend the time required for membrane wetting. The main methods are membrane material modification, absorbent improvement, and membrane structure improvement. This review introduces the mechanisms of membrane wetting and summarizes three types of wetting. The differential equations in the wet state and the boundary conditions needed to establish a model for the absorption mechanism will allow future researchers to study membrane wetting more effectively.

## 2. Membrane Contactor Operating Principle and Wetting Mechanism

### 2.1. Operating Pathway

**Figure 2:**
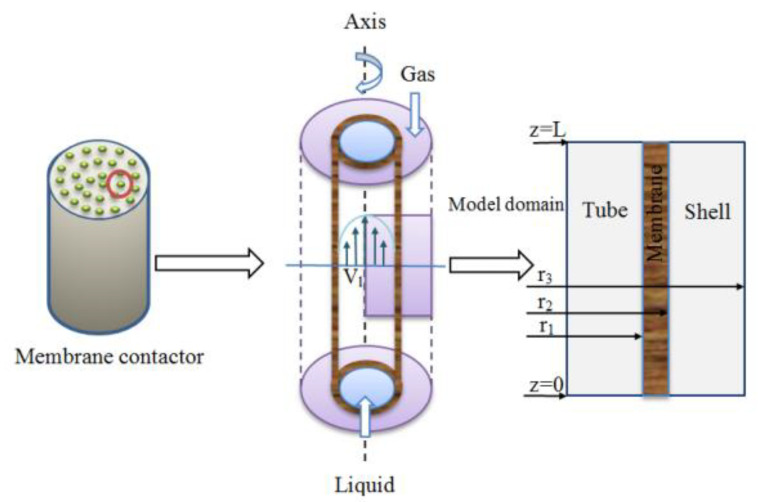
Operating principle of membrane absorption method [5].

In gas separation, the most widely used membrane module, i.e., hollow fiber membrane, has become a popular research topic. Herein, a hollow fiber membrane is used as an example to introduce its operating principle and wetting mechanism. The membrane contactor involves three processes for separating gas: a. the separated gas in the mixed gas is driven by the concentration difference to reach the membrane hole; b. the gas is transmitted to the gas–liquid interface through membrane pore diffusion; c. the gas crosses the gas–liquid interface and reacts with the liquid phase. The operating principle is shown in Figure 2.

When removing typical gases, the mass transfer resistance of the membrane contactor includes liquid-phase, gas-phase, and membrane resistances. According to the principle of parallel resistance, the total mass transfer resistance formula is as shown in Equation (1) [23]:

(1) $$\frac{1}{K_{G}}=\frac{1}{k_{g}}+\frac{1}{k_{m}}+\frac{1}{m\beta k_{1}}$$

where $\frac{1}{K_{G}}$ is the total mass transfer resistance; $\frac{1}{k_{g}}$ is the gas phase resistance; $\frac{1}{k_{m}}$ is the membrane resistance; m is the liquid-phase mass transfer coefficient; β is the phase equilibrium constant; and $k_{1}$ is the chemical enhancement factor.

### 2.2. Wetting Mechanism

**Figure 3:**
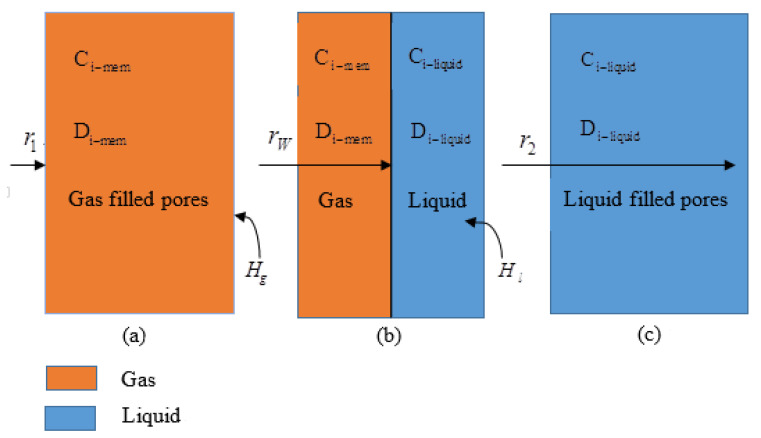
Schematic diagram of three wetting states. (**a**) Non-wetted, (**b**) partially wetted, and (**c**) fully wetted conditions.

A large number of studies have shown that a slight membrane wettability can cause the membrane phase resistance to increase rapidly, which consequently increases the total mass transfer resistance and causes a significant decrease in mass transfer rate [24,25,26,27,28,29,30,31]. Rangwala et al. indicated that when the membrane has a small degree of wetting (<2%) [32], the membrane resistance will increase rapidly, accounting for approximately 60% of the total mass transfer resistance. Therefore, preventing or reducing the wetting of the membrane contactor during operation is key to the industrialization of the membrane contactor. The wetting of membrane pores is an important factor hindering the development of membrane absorption methods. There are three cases of membrane wetting, as shown in Figure 3, which are non-wetting, partial wetting, and full wetting. 

#### 2.2.1. Non-Wetting Mode

When a membrane is not wetted, the gaseous phase directly passes through the membrane pores. During this time, the membrane pores are filled with the gaseous phase, the mass transfer rate is the highest, and the reaction rate is the fastest, as shown in Figure 3a. Generally, researchers in this field assume the model to be in this state when studying the effects of related parameters. The simplified overall equation is given by Equation (2):

(2) $$-\left(\frac{1}{r}\frac{\partial \left(r{\left(C_{i}V\right)}_{r}\right)}{\partial r}+\frac{1}{r}\frac{\partial {\left(C_{i}V\right)}_{\theta }}{\partial \theta }+\frac{1}{r}\frac{\partial {\left(C_{i}V\right)}_{Z}}{\partial Z}\right)-\left(\frac{1}{r}\frac{(r\left(\partial J_{i}{)}_{r}\right)}{\partial r}+\frac{1}{r}\frac{{\left(\partial J_{i}\right)}_{\theta }}{\partial \theta }+\frac{{\left(\partial J_{i}\right)}_{Z}}{\partial Z}\right)+R_{i}=0$$

The material balance in the membrane considers only the diffusion of the gas within the membrane pores, expressed as Equation (3) [33,34]:

(3) $$\frac{\partial C_{i\text{-}mem}}{\partial t}+D_{i\text{-}mem}\left[\frac{\partial^{2}C_{i\text{-}mem}}{\partial r^{2}}+\frac{1}{r}\frac{\partial C_{i\text{-}mem}}{\partial r}+\frac{\partial^{2}C_{i\text{-}mem}}{\partial z^{2}}\right]=0$$

where $D_{i-mem}$ and $C_{i-mem}$ are the molecular diffusivity and concentration distribution of component i in the gas phase within the membrane, respectively. 

#### 2.2.2. Partially Wetted Mode

For the partially wetted mode, there are two phases in the membrane pores—the gas-filled and liquid-filled phases—as shown in Figure 3b. In the gas-filled phase, the material balance can be derived using Equation (4) [33,34]:

(4) $$D_{i,g\text{-}mem}(\frac{\partial^{2}C_{i,g\text{-}mem}}{\partial r^{2}}+\frac{1}{r}\frac{\partial C_{i,g\text{-}mem}}{\partial r}+\frac{\partial^{2}C_{i,g\text{-}mem}}{\partial z^{2}})=0$$

In the case of partial wetting, the mass transfer equation of the liquid phase on the membrane side is given by Equation (5):

(5) $$D_{i,l\text{-}mem}(\frac{\partial^{2}C_{i,l\text{-}mem}}{\partial r^{2}}+\frac{1}{r}\frac{\partial C_{i,l\text{-}mem}}{\partial r}+\frac{\partial^{2}C_{i,l\text{-}mem}}{\partial z^{2}})=0$$

#### 2.2.3. Fully Wetted Mode

The fully wetted model implies that the liquid phase completely fills the membrane pores. At this time, all the membrane pore volumes are occupied by the liquid, and the mass transfer rate is the lowest, as shown in Figure 3c. In this model, the material balance can be obtained from Equation (6):

(6) $$D_{i,l\text{-}mem}(\frac{\partial^{2}C_{i\text{-}mem}}{\partial r^{2}}+\frac{1}{r}\frac{\partial C_{i\text{-}mem}}{\partial r}+\frac{\partial^{2}C_{i\text{-}mem}}{\partial z^{2}})=0$$

where $D_{i,l-mem}$ and $C_{i-mem}$ are the molecular diffusivity and concentration of component i in the liquid-filled phases within the membrane, respectively. 

When the membrane pores are fully wetted, the penetration pressure of the solution in the liquid phase region is the minimum pressure at which the liquid absorbent enters the membrane pores. Its value can be evaluated by Laplace’s equation (Equation (7)):

(7) $$LEP=\frac{-4\gamma \cos\theta }{d_{\max}}$$

where γ is the surface tension of absorbent, θ is the contact angle between the solvent and membrane surface, and $d_{\max}$ the inner diameter of the largest membrane pore. Therefore, methods for preventing the wetting of the film include using a solvent with surface tension, or a method using a hydrophobic film material. The wetting of the membrane material begins at larger membrane pores. As time progresses, the smaller membrane pores gradually begin to wet until they are completely wet, as shown in Figure 3c. Therefore, it is extremely important to obtain a film material with a large contact angle and a solvent with a large surface tension to resist membrane wetting.

## 3. Influencing Factors of Gas Separation in Membrane Contactors

### 3.1. Membrane Material

The membrane material acts as a barrier that separates the gas phase from the liquid phase and has no selectivity. It primarily depends on the gas-phase selective absorption by the liquid-phase absorbent to achieve a mixed gas separation. The structure and properties of the membrane material determine the maximum membrane pore size and the two-phase contact angle. Since most of the absorbents used for separation and other gases are organic solvents, the surface tension of organic solvents is often low, which is prone to problems such as membrane wetting and increased mass transfer resistance. To solve these problems, many scholars have carried out improvements to membrane materials, and have achieved many results. The following sections describe the research progress of polymer membranes and ceramic membranes.

#### 3.1.1. Research Progress of Polymeric Membrane

Membranes are commonly classified into polymeric and ceramic membranes according to the material type [35]. Polymer membranes mainly include polysulfones, polyamides, polyimides, polyesters, etc. Organic membranes are widely used due to their wide range of materials, low manufacturing costs, and high assembly density of membrane components. Several works have been carried out to improve the performance in terms of membrane materials [36,37,38]. The main problems are still high performance, membrane wetting, and membrane fouling. 

The methods of processing the surface of the membrane mainly include blending modification, chemical grafting, surface coating, etc. The treated membrane has better separation performance and anti-wetting properties. Naim et al. added nonsoluble additives, such as lithium chloride, methanol, and phosphoric acid (PA) to a porous asymmetric polyetherimide (PEI) membrane to improve its physical properties [39]. Xu et al. fabricated an inorganic–organic fluorinated titania–silica (fTiO_2_–SiO_3_)/PVDF composite membrane via an in situ vapor-induced hydrolyzation process, incorporated with perfluorodecyltriethoxysilane (PFTS) grafting, followed by a hydrophobic modification. They found that the fabricated membrane exhibited a high CO_2_ absorption flux of 8.0 × 10^−3^ mol m^−2^ s^−1^ compared to a pristine PVDF membrane, and it possessed high chemical resistance and hydrophobicity [40]. Ma et al. addressed the problem of membrane materials getting easily wetted owing to long-term soaking in chemical absorbents by grafting commercial PVDF membranes with an organosilane on the surface [41]. Chen et al. added hydrophobic materials to the inner coating to densify the inner layer; the more hydrophobic the inner coating is, the higher the viscosity of the coating. This delays the phase transition process and improves the wettability of the membrane [42]. Lee et al. modified the hydrophobicity of the surface by binding fluoroalkylsilane (FAS) with OH-groups and deploying hydrophobic chains on the opposite side [43]. 

#### 3.1.2. Research Progress of Ceramic Membrane

As we all know, the price of polymer membrane is cheap, but it has to be replaced frequently due to swelling, which increases the operating cost. So, recent studies have focused on gas–liquid membrane contactors composed of a ceramic material with excellent thermal chemical stability and extraordinary mechanical strength [44,45,46,47]. 

Ceramic membranes have attracted attention because of their high separation efficiency, good chemical stability, acid and alkali resistance, organic solvent resistance, bacterial resistance, high temperature resistance, and pollution resistance. Common fabrication techniques of ceramic membranes include extrusion, pressing, slip casting, tape casting, sol–gel, phase inversion, anodic oxidation, and chemical vapor deposition (CVD) methods [35]. Currently, ceramic hollow fiber membranes are fabricated by combined phase-inversion/sintering in an asymmetric structure with unique finger-like and sponge-like structures [47]. Lee et al. fabricated ceramic hollow fiber membrane contactors (CHFMCs) using a phase inversion/spinning method, and the module consisted of 200 ceramic hollow fibers and was tested in different working conditions [48]. 

Due to having superior surface chemical properties compared to polymer membranes, ceramic membranes have become very common in the field of sewage treatment. Some researchers have also used them for gas separation in harsh environments. Ogunlude et al. used ceramic nanostructured membranes to separate gas from biogas, and the separation of CH_4_ from CH_4_/CO_2_ has achieved good performance, and the treated biogas can be used as alternative energy [49]. An et al. fabricated a superhydrophobic ceramic (SC) membrane contactor for capturing CO_2_ from coal-fired power plant flue gas. They found that the detrimental effects of wetting can be alleviated by periodic drying, and used two SC membrane contactors alternatively operated with periodic drying to ensure continuous CO_2_ removal with high efficiency [50].

**Figure 4:**
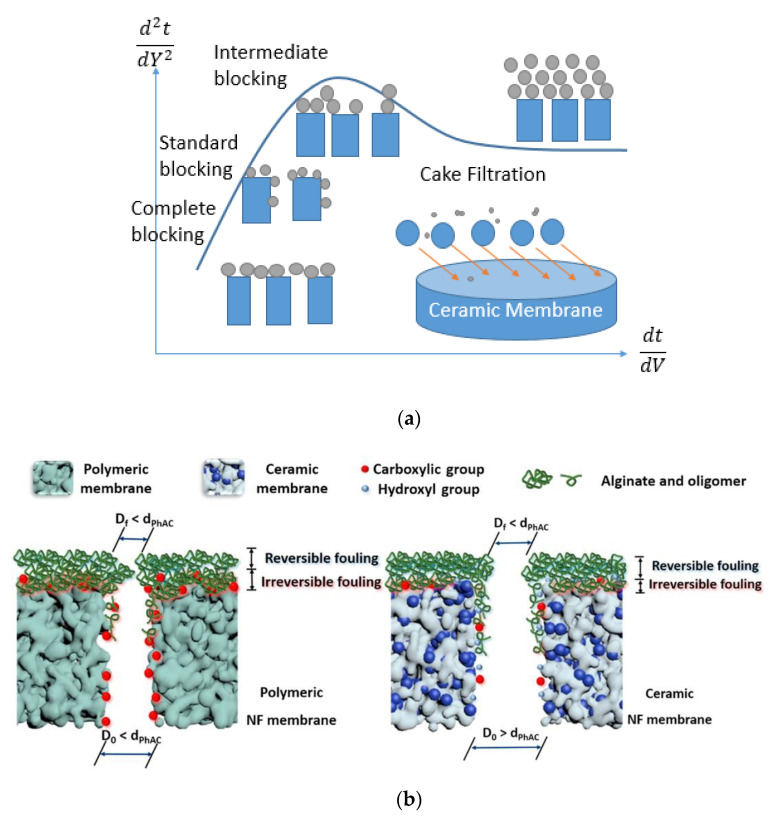
Schematic illustration of (**a**) fouling characteristics of ceramic membranes using filtration models [55] and (**b**) fouling of polymeric and ceramic nanofiltration membranes by alginate [56].

For the wide application of membrane contactors, membrane fouling has been the bottleneck since the birth of membrane separation technology. There have been many reports on the membrane fouling mechanisms and fouling control strategies of polymers [51,52]. Changes in polymer membrane selectivity and flux of polymer membranes can be attributed to membrane fouling and microbial invasion, and concentration polarization and free chlorine attack [53]. However, there are no clear explanations of the membrane fouling mechanism of ceramic membranes [54]. Lee et al. employed the filtration models to analyze the fouling characteristics of ceramic membranes, which have been applied to polymeric membranes. They indicated that these models built on cake filtration could also be utilized to simulate natural organic matter (NOM) fouling of ceramic membranes, as shown in Figure 4a [55]. Zhao et al. elucidated the removal mechanisms governing pharmaceutically active compounds (PhACs) using four types of commercially available ceramic nanofiltration (NF) membranes and suggested that electrostatic interactions were more dominant than hydrophobic interactions due to higher hydrophilicity and lower densities of COOH and OH groups on the ceramic surface compared to the polymeric counterparts, as shown in Figure 4b [56]. However, this was only applicable when PhACs were spiked in deionized water. Hydrophobic interactions and adsorption played a major role when real wastewater effluent was used to form a fouling layer [35]. Therefore, further research on the mechanism governing the separation and fouling processes of ceramic NF membranes or fine UF membranes in real water treatment is needed.

Table 1 lists the uses of different membrane materials for gas removal processes.

### 3.2. Absorbents

An absorbent is a solvent used in membrane contactors for chemical reactions with mixed gases to achieve gas separation. The selection of absorbent is crucial; properties such as recyclability, high surface tension, volatility, easy regeneration, being environmental friendly, and absorption efficiency must be considered [62]. Typical absorbents include monoethanolamine (MEA), diethanolamine (DEA), and methyldiethanolamine (MDEA). However, although alcohol amine solvents have the advantage of facilitating a fast absorption rate, the amine solutions have notable defects, such as thermal and oxidative degradation, equipment and pipeline corrosion, toxicity, and, most importantly, easy wetting of the membrane owing to the a low surface tension of the absorbent [63,64]. Therefore, researchers have been actively exploring various absorbents and their characteristics. Some of the main absorbents and their characteristics relevant to the current research are introduced below. 

#### 3.2.1. Amine Solvent

As alcohol amine solvents have a high capacity for gas absorption, and they have the characteristics of fast absorption rate, large absorption capacity, and simple regeneration. Currently, amine solvents are commercially available, and widely used and studied in practical applications. An alcohol amine solution developed from a single absorbent to a mixed absorbent improves the gas removal performance of a membrane. As there are a large number of literature reviews that have introduced the related research, it is not extensively covered here. However, the knowledge on active alcohol ammonium solvents is still relatively small. The following section introduces some of the latest developments in active alcohol ammonium solvents. 

#### 3.2.2. Activated Alcohol Amine Solvent

The activated alcohol amine solvent is an improved solvent by adding an activator to the original ammonia solution. In recent years, to improve the CO_2_ absorption performance, researchers have studied the mixing of active alcohol ammonium and alcohol amine solvents. Rahmawati et al. added piperazine (PZ) and monosodium glutamate (MSG) as activators at a 1% *w*/*w* concentration in a MDEA 30 wt% solution to form an aqueous solution of activated MDEA. Their results showed that MDEA-PZ was the best absorbent for CO_2_ with a separation efficiency 1.4 times higher than that of an inactive MDEA absorbent [65]. Saidi et al. studied the mass transfer of 1-dimethylamino-2-propanol as a new type of amino alcohol solvent in a split absorption tower-stripping unit. Their modeling indicated that the CO_2_ absorption flux and the overall mass transfer coefficient ($k_{G}a_{V}$) for CO_2_ absorption in different solutions could be ranked as follows: PZ > MEA > DEA > 4-diethylamine-2-butanol (DEAB) > 1-dimethylamino-2-propanol (1DMA2P) > MDEA > triethanolamine (TEA) [66]. These studies on active alcohol amine solutions had a positive impact on the application and development of alcohol amine solvents. However, more experimental verification and long-term running tests are needed for applying the above in industrial production.

#### 3.2.3. Amino Acid Solutions

Amino acid solutions have the same functions as alcohol amine solvents; however, they also have other advantages. For example, they possess better oxidation stability and resistance to degradation, and can be nonvolatile after adding salt. As a result, they have proven themselves to be promising absorbents [67]. Yan et al. used a natural amino acid, potassium L-argininate (PA), as an absorbent in HFMCs to absorb CO_2_, and found that the CH_4_ content in biogas treated with PA could reach up to 99.15% [34]. Compared with MEA, it has better applicability; furthermore, the PA solvent can use a lower solvent concentration, lower liquid velocity, and higher reaction temperature [68].

To achieve a higher reaction rate, the amino acid salt solution is often used in conjunction with other solutions. Jian et al. added PZ to a glycine solution and compared it with a single glycine acid solution. They found that the CO_2_ capture performance of the active glycinate solution was significantly better than that of the inactive glycinate solution. The average value of reaction rate in the activated solution was 1.3 times that of the inactive solution [69]. The amino acid solution can also be mixed with a salt solution (e.g., K_2_CO_3_, K_2_B_4_O_7_), to obtain many beneficial effects. Li et al. mixed lysine and potassium carbonate to absorb CO_2_. Their results showed that the optimal molar ratio of the K_2_CO_3_ solution was 5:5 (the total concentration of the absorbent was 1 M), which was 1.68 times the absorption load of the pure K_2_CO_3_ solution [70]. Feng et al. mixed arginine with potassium carbonate and the results showed that L-arginine not only significantly increased the rate of CO_2_ absorption by the potassium carbonate solution, but also increased the desorption rate by two-fold. Under the same conditions, the normalized mass transfer flux of 0.1 M L-arginine activated potassium carbonate solution to absorb CO_2_ increased by approximately 85% [71]. Lu et al. added potassium borate as an activator to glycinate (GLY) aqueous solution to form an activated composite absorbent (ACA) for capturing CO_2_, and studied the effect of activator concentration, operating temperature, and adsorption flow rate on the total volume mass transfer. It was found that the CO_2_ capture efficiency of ACA was significantly higher than that of the GLY aqueous solution. It was also observed that the activation effect of a small amount of activator on the ACA was greater than that of a large number of activators [72].

#### 3.2.4. K_2_CO_3_

Compared with traditional solutions, K_2_CO_3_ solution shows better thermal stability and lower regeneration costs, and can be considered as an appropriate absorbent alternative for CO_2_ separation. It is thermally more stable than alcohol amine solutions and can be employed in CO_2_ sequestration processes at high temperatures. The low regeneration cost of K_2_CO_3_, particularly at higher CO_2_ concentrations, makes it a reactive stripping absorbent for efficient CO_2_ capture from gaseous streams. In addition, the K_2_CO_3_ aqueous solution is non-organic, and has a high surface tension and low tendency for wetting the membrane contactor pores compared to alkanolamine absorbents. These advantages strongly justify the utilization of this absorbing solution [73].

A gas source typically contains more than a single gas. For example, untreated natural gas contains CO_2_, H_2_S, CH_4_, and other gases. It is necessary to remove all the gases at the same time. The requirements for the absorbent for the removal of two or more gases are often very high, and the absorbent is required to achieve a relatively high removal rate for all the gases. A single K_2_CO_3_ solvent is effective in removing H_2_S; however, its performance in removing CO_2_ is not very considerable. Although the reaction kinetics of K_2_CO_3_ and CO_2_ are very slow, amino acid salts can promote the chemical reaction of K_2_CO_3_. In addition, compared with amine solutions such as MEA, amino acid salts consume less renewable energy, which can reduce the cost of CO_2_ regeneration [5]. Therefore, researchers have investigated mixing these two types of absorbents with process gases.

**Figure 5:**
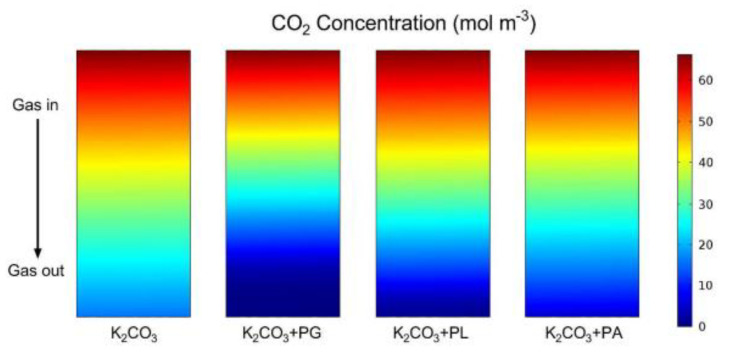
CO_2_ concentration distribution in the tube with different absorbents (absorption temperature: 313 K; operating pressure: 4 bar; gas flow rate: 800 mL/min; and liquid flow rate: 75 mL/min) [74].

Li et al. added amino acid salts to a K_2_CO_3_ solution. Their results showed that the use of amino acid salts (AASs) in the K_2_CO_3_ solution as an accelerator could significantly improve the CO_2_ absorption performance. They also compared the decarbonization effects of three amino acids. Of these, K_2_CO_3_ and potassium glycinate (PG) was the most effective, as shown in Figure 5 [74]. Zhang et al. found that the decarburization effect after adding potassium lysinate (PL) solvent to K_2_CO_3_ was better than that of a single K_2_CO_3_ solvent and concluded that the gas phase is the most important influencing factor [5]. 

#### 3.2.5. Nanofluids

**Figure 6:**
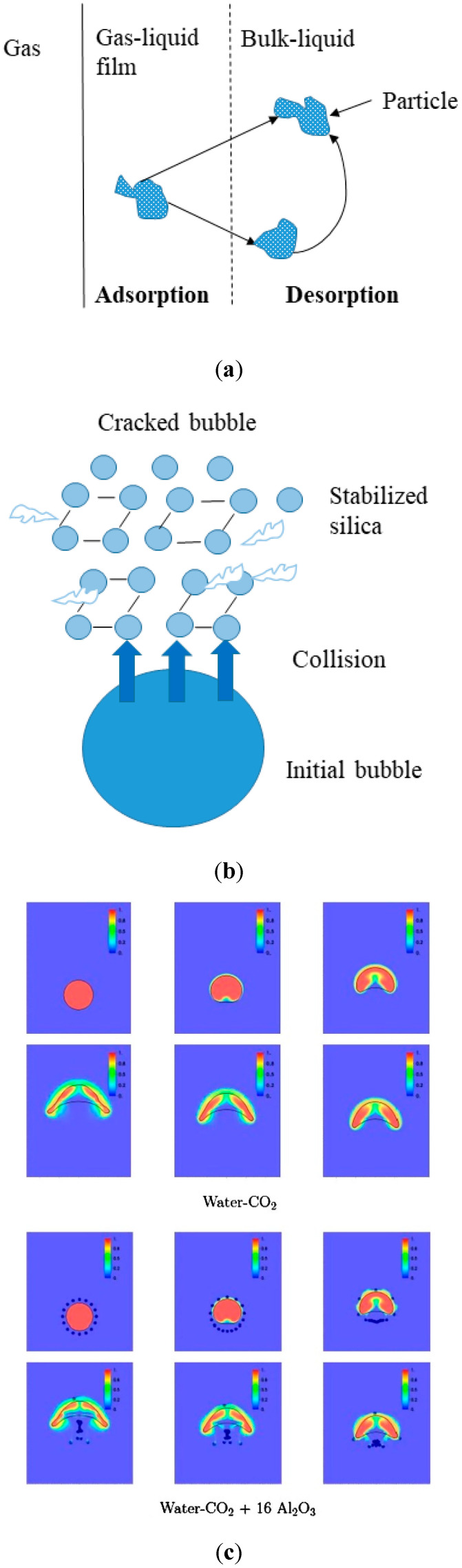
(**a**) Grazing (or shuttle) effect [78]; (**b**) mass transfer interface increase by bubble breaking effect [79]; (**c**) gas–liquid interface shape in hydrodynamic effect [80].

Nanofluids are nanomaterials with a diameter of less than 100 nm and can be stably suspended in the liquid [75]. Nanofluids act as absorbents, causing the laminar flow of the traditional membrane contactor system to become turbulent [76]. Among the many nanoparticles used to prepare nanofluids, Al_2_O_3_, SiO_2_, and CuO are the most common. Pang et al. provided a comprehensive review of the mass transfer characteristics of nanofluids [77]. Three possible mechanisms for enhancing the mass transfer of nanofluids were proposed in the literature, which include (a) grazing (or shuttle) effect [62], (b) fracture effect due to bubbles [78], and (c) gas–liquid interface shape in hydrodynamic action [79], which increases the mass transfer interface. The schematic diagrams of these mass transfer mechanisms are illustrated in Figure 6.

In recent years, numerous studies have confirmed that nanofluids could significantly enhance the mass transfer rate. However, the inconsistencies among these research results and the lack of reliable mechanisms to explain these conflicting results indicate that more research is needed to clarify the impact of nanoparticles of different types on mass transfer in applications. Tinge et al. proposed a model considering the shuttle or grazing effect theory. The particles were supposed to transport an additional amount of gas to the liquid bulk through adsorption in the gas–liquid diffusion layer and desorption in the liquid bulk, which resulted in a larger mass transfer coefficient [78]. Kim et al. proposed the bubble breaking model to explain the anomalous enhancement in mass transfer with visualization experiments. They reported that the detachment of the gas bubbles in nanofluids occurred during a much shorter period of time than in the base fluid; the faster detachment led to a higher frequency rate, as reported in the literature, thus yielding a smaller bubble size with an increased bubble quantity [79]. Yoon et al. demonstrated the two-film theory: the gas–liquid film interface controls resistance to the transfer of a material from one phase to another. It diffuses through the gas film and proceeds at a rate that is proportional to the difference between solute concentrations in the gas on the outside and inside of the gas film, while the diffusion through the liquid film is controlled by the difference between the concentration of solute in the liquid at the interface and its concentration on the other side of the liquid film [80]. According to these three explanations, when the nanoparticle concentration increases, the mass transfer rate should increase. However, several studies showed that when nanoparticles increased to a certain extent, the mass transfer showed a tendency to decrease [81,82,83]. Therefore, more studies are needed on the mass transfer mechanism of nanofluids.

Krishnamurthy et al. studied the effect of water-based nanofluids containing 20 nm Al_2_O_3_ nanoparticles on the mass diffusion of fluorescent dyes and found that the diffusion coefficient in nanofluids was 13 times that of water without the nanoparticles [84]. Ansaripour et al. also confirmed this finding by using α-Al_2_O_3_/water and γ-Al_2_O_3_/water nanofluids with a nano-porous hollow fiber dialysis membrane contactor to remove CO_2_ from N_2_/CO_2_ gas mixtures. The results showed that the increases in the removal efficiency of α-Al_2_O_3_/water and γ-Al_2_O_3_/water nanofluids with a concentration of 0.02 vol% in comparison with distilled water were 12.2% and 21.6%, respectively [85]. Olle et al. used functional magnetic nanoparticles to enhance oxygen mass transfer and found that it was increased up to six-fold by a water-based Fe_3_O_4_ nanofluid [86]. These studies have proved the enhancement effect of nanoparticles on mass transfer and gas removal. However, some studies have not achieved positive results. Sumin et al. obtained contradictory results. They found that micron-sized Al_2_O_3_ had no effect. While nano-Al_2_O_3_ enhances the weak absorption of CO_2_, activated carbon (AC) and carbon nanotube (CNT) particles effectively improve the gas–liquid mass transfer [87]. Therefore, although current research results usually show that these particles benefit the gas separation, it is still difficult to judge their performance in large-scale applications, and more research is needed in this area. For example, the role of nanoparticles in the presence of chemical absorbents is still questionable.

To date, there have been some studies suggesting that nanofluids have certain anti-wetting properties. Talavari et al. found from a long-term absorption test that membranes showed a tendency toward non-wetting, and the membrane pores in a nanofluid absorbent were not blocked [88]. However, their study lacked theoretical support, and there were no comparative experiments to substantiate their claims. Therefore, it remains to be verified whether nanofluids exhibit resistance to moisture.

The characteristics of these absorbents are shown in Table 2.

### 3.3. Contactor Structure

**Figure 7:**
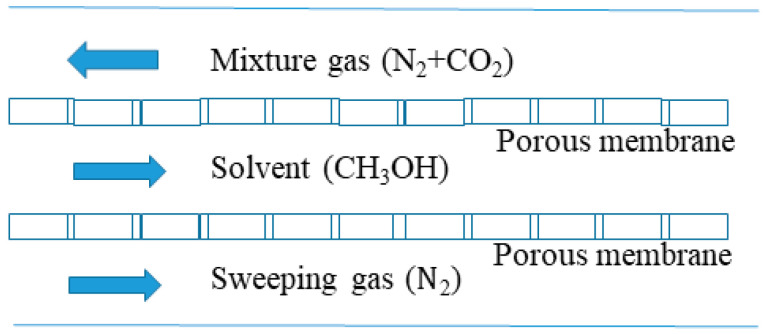
Schematic arrangements of membranes in the proposed novel dual-membrane system [100].

It is more typical to increase the separation rate by changing the internal structure of the membrane contactor. Generally, a baffle is added inside the membrane contactor to achieve the second reflux of the gas, or a double-sleeve structure is used to improve the absorption efficiency. The occurrence of some undesirable phenomena in the separation process can be reduced by using shell ditch flow. Zhang et al. discovered that adding baffles in the membrane contactor can effectively reduce the shell-side groove flow phenomenon often discovered in experiments [97]; additionally, it can promote the liquid-phase flow in the tube and the two-phase mass transfer along the membrane, thereby improving the separation effect of membrane packing. Yang et al. used seven methylsiloxane-coated hollow fiber membranes as structural fillers. In the ethanol–water solution separation, two baffles were installed on the shell side of the membrane contactor [98]. The results show that the baffle assists the membrane module in obtaining a higher mass transfer coefficient and a smaller mass transfer resistance, especially at higher steam speeds. Zhang et al. adopted an improved double-sleeve hollow fiber membrane contactor for removing ammonia from an aqueous solution under a low-absorption liquid concentration and a low-absorption liquid flow rate [99]. The overall mass transfer coefficient of this improved double-sleeve hollow fiber membrane contactor is much higher than that of the single membrane module; under the same experimental conditions, its special configuration reduces the dilution of the byproduct ammonium salt solution by osmotic distillation and the byproduct ammonium sulfate. The concentration is approximately 9% higher than that of the single membrane module. Wang et al. studied the concept of a dual membrane system, in which a second membrane was added to the single membrane system [100], and a purge gas or a lower applied pressure on the permeate side of the porous or nonporous secondary membrane. The effect of a double film was analyzed theoretically. It is theoretically believed that the new model will obtain better absorption liquid regeneration effects and higher gas removal efficiency, as shown in Figure 7.

### 3.4. Others

In the membrane contactor, other parameters affect gas separation. For example, increasing the module length, membrane porosity, gas flow rate, liquid flow rate, and other parameters can change the gas separation efficiency. In actual production, reasonable parameters will significantly improve gas separation efficiency. The parameters affecting the separation performance of the membrane contactor are discussed below.

**Figure 8:**
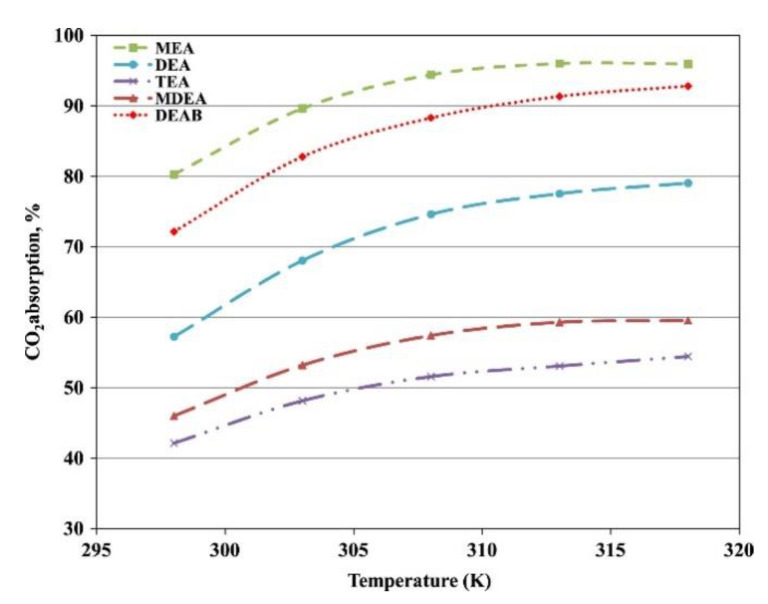
Effect of temperature on CO_2_ absorption for various absorbents under the non-wetting condition (CO_2_: 20%; N_2_: 80%; gas velocity: 0.2 m/s, liquid velocity: 0.01 m/s; absorbent concentration: 1 M; pressure: 120 kPa) [107].

**Figure 9:**
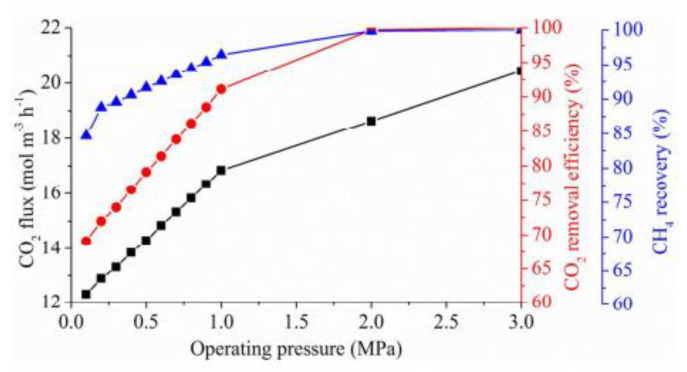
Effect of operating pressure on the hollow fiber membrane contactor (HFMC) performance for various absorbents (initial CO_2_ concentration: 40%; PA concentration: 5 wt%; gas velocity: 0.32 m/s; liquid velocity: 0.06 m/s) [108].

A large number of studies have shown that increasing the absorbent flow rate and concentration, reducing the gas flow rate, and reducing the volume fraction of the separated gas in the mixed gas are conducive to gas purification [10,101,102,103,104]. When the flow rate of an absorbent is increased, the diffusion rate of the liquid phase will increase. This is due to the increase in the liquid flow rate, which increases the pressure difference between the gas and liquid phases, and further increases the reaction rate. Tahvildari et al. established a gas–liquid chemical reaction and mass transfer model using 2-amino-2-methyl-1-propanol (AMP) as an absorbent in HFMCs. The results obtained by the effective element method were consistent with the experimental data: increasing the solvent flow rate can promote the removal of the hollow fiber membrane contactor pairs; the countercurrent process was more conducive to achieving the highest separation efficiency [105]. Nakhjiri et al. discovered that increasing the operating parameters, such as module length, membrane porosity, and absorbent concentration, can increase the removal rate in the gas mixture [106], however, the increase affected the separation efficiency negatively. Majid found that increasing the temperature enhanced the CO_2_ absorption and, although increasing the temperature decreased the CO_2_ solubility in the liquid phase, it improved the reaction rate and diffusion coefficients, as illustrated in Figure 8 [107]. Zhang et al. also studied the effect of gas pressure on the removal efficiency and flux, as shown in Figure 9 [108]. It can be seen that as the pressure increased, both the removal efficiency and CO_2_ flux increased.

Studies based on the influences of membrane materials, absorbents, and membrane structures are important for evaluating the gas separation performance in membrane contactors, which can provide important judgments for the future research and development of membrane contactors. 

## 4. Conclusions and Future Perspectives

This review primarily summarizes the working principle of a membrane contactor and the differential equations under the three states of membrane wetting. It also introduces the state of the art in membrane materials and the main improvement methods of membrane materials, including blending modification, surface coating, and surface treatment. These three methods differ in their characteristics and applications. With regard to absorbents, this review enumerates some widely used absorbents, summarizes their characteristics, and discusses the shortcomings of previously published papers in this field. Moreover, this study presents the structure effect of a membrane contactor on gas separation. Modifications to the membrane structure are helpful in improving the gas separation efficiency; however, large-scale commercial applications need to be further investigated. The influence of other parameters is also very significant for further research in this field, specifically, those that serve as index parameters for judging the quality of the absorbents, membrane materials, and membrane structures.

The direction of the future development of membrane contactors is still the improvement of membrane materials and the renewal of absorbents, because membrane wetting is still the main obstacle of the membrane gas absorption method. In addition, in the process of comprehensive research on practical applications, the evaluation of economic benefits can be added to the process, and the most common recycling systems should also be discussed and studied. This helps to achieve practical applications of the membrane contactors faster. In a word, the membrane contactor is a promising gas separation tool and deserves more attention.

## Figures and Tables

**Table 1:** Summary of different membrane materials used for gas removal.

Membrane Material	Gas	Absorbent	Highlight	Ref
Hydrophilic membrane	Sulfur dioxide	NaOH	The hydrophilic membrane contactor exhibited superior selectivity up to 114.	[57]
Ceramic hollow fiber membrane	Flue gas	Monoethanolamine (MEA)	Process applicability and stability of the ceramic hollow fiber membrane module were both confirmed.	[58]
Dual-layer PVDF SiO_2_ composite membrane	Flue gas	Diethanolamine (DEA)	Advantages of high gas permeability structure as well as high hydrophobicity.	[42]
Porous hydrophobic modified ceramic hollow fiber membrane	Carbon dioxide	30 wt% MEA	The novel technique reduces the stripping energy.	[59]
Hybrid polyvinylidene fluoride-hexadecyltrimethoxysilane (PVDF–HDTMS) membrane	19 vol.% carbon dioxide	1 mol/L DEA	The membrane showed excellent long-term CO_2_ flux in the membrane contactor.	[59]
Hydrophobic PP/CH_3_SiO_2_ composite hollow fiber membrane	20 vol.% carbon dioxide	30 wt% MEA	CO_2_ absorption flux of the nanocomposite membranes was stable within 30 days operation time.	[60]
Supported ionic liquid membranes (SILMs)	Carbon dioxide/oxygen	MEA	Remarkable mechanical properties and hydrophobicity for membranes.	[61]

**Table 2:** Types and characteristics of absorbents.

Absorbent Type	Main Solvents	Advantages	Disadvantages	Ref.
Alcohol amine solvent	MEA, DEA, TEA, MDEA, PZ	The characteristics of fast absorption rate, large absorption capacity and simple regeneration	Easily cause corrosion to equipment, easy to degrade and easy to wet	[89]
	DEAB	Low energy consumption for degradation		[90]
	1-dimethylamino-2-propanol (1DMA2P)	High CO_2_ absorption capacity, regeneration energy of 1DMA2P is lower than that of MDEA, DEA, MEA, and PZ	High volatility of DMAPA and substantial energy requirement	[91]
	3-Dimethylaminopropylamine (DMAPA)	The high removal efficiency and CO_2_ cyclic capacity		[92]
Nanofluid absorbent	Al_2_O_3_	Increased diffusion coefficient and increased reaction rate	High viscosity and high cost	[88]
	SiO_2_			
K_2_CO_3_	K_2_CO_3_	High surface tension with low tendency to membrane wetting thermal stability, low regeneration cost	High energy consumption, high heat capacity, strong corrosive	[93]
Amino acid solutions (AASs)	Potassium L-argininate (PA)	Environmentally friendly, better affinity, lower solvent concentration, lower liquid velocity, and higher reaction temperature can be used	The reaction rate with CO_2_ is slow, generally mixed with other solutions for use	[93]
	Potassium lysinate (PL)	High gas absorption capacity, and has a higher chemical reactivity to CO_2_		[87]
	Potassium sarcosine (PS)	Thermal stability and easy regeneration		[94]
	Potassium glycinate (PG)	High reactivity toward CO_2_ and less regeneration energy consumption		[95]
Ionic liquids (ILs)	\	Negligible volatility, high thermal stability, adjustability, solvation, high CO_2_ solubility	High cost, high viscosity, and high energy consumption in the regeneration process	[96]
